# A Steiner tree-based method for biomarker discovery and classification in breast cancer metastasis

**DOI:** 10.1186/1471-2164-13-S6-S8

**Published:** 2012-10-26

**Authors:** Md Jamiul Jahid, Jianhua Ruan

**Affiliations:** 1 Department of Computer Science, The University of Texas at San Antonio, One UTSA Circle, San Antonio, TX 78249, USA; 2 Cancer Therapy and Research Center, The University of Texas Health Science Center, San Antonio, TX 78229, USA

## Abstract

**Background:**

Metastatic breast cancer is a leading cause of cancer-related deaths in women worldwide. DNA microarray has become an important tool to help identify biomarker genes for improving the prognosis of breast cancer. Recently, it was shown that pathway-level relationships between genes can be incorporated to build more robust classification models and to obtain more useful biological insight from such models. Due to the unavailability of complete pathways, protein-protein interaction (PPI) network is becoming more popular to researcher and opens a new way to investigate the developmental process of breast cancer.

**Methods:**

In this study, a network-based method is proposed to combine microarray gene expression profiles and PPI network for biomarker discovery for breast cancer metastasis. The key idea in our approach is to identify a small number of genes to connect differentially expressed genes into a single component in a PPI network; these intermediate genes contain important information about the pathways involved in metastasis and have a high probability of being biomarkers.

**Results:**

We applied this approach on two breast cancer microarray datasets, and for both cases we identified significant numbers of well-known biomarker genes for breast cancer metastasis. Those selected genes are significantly enriched with biological processes and pathways related to cancer carcinogenic process, and, importantly, have much higher stability across different datasets than in previous studies. Furthermore, our selected genes significantly increased cross-data classification accuracy of breast cancer metastasis.

**Conclusions:**

The randomized Steiner tree based approach described in this study is a new way to discover biomarker genes for breast cancer, and improves the prediction accuracy of metastasis. Though the analysis is limited here only to breast cancer, it can be easily applied to other diseases.

## Background

The identification of marker genes involved in cancer is a central problem in system biology. Many studies have used gene expression data for marker identification in breast cancer and other diseases [1,2]. However, noisy data, small sample sizes, and heterogeneous experimental platforms make the marker selection procedure difficult and dataset-specific. As a result, different studies on the same disease often have very few gene markers in common. For example, two studies [3,4] identified 70 and 76 gene marker for breast cancer, which were also validated later by two other studies [5,6], but they have only three genes in common.

To improve the stability of marker selection, other complementary genomic information such as pathways has been used [7-9]. The problem of pathway-based approach, however, is that the majority of human genes are not assigned to a specific pathway [10]; therefore there is a strong possibility that a true marker may be out of consideration for not being assigned to a pathway. To circumvent this problem, [10] proposed to incorporate protein-protein interaction (PPI) networks for discovering small sub-networks, which may represent novel pathways, as potential markers. They found that such subnetwork-based markers can both improve classification accuracy and increase cross-dataset stability. Other studies have attempted to use gene co-expression networks or hybrid networks developed from various sources instead of PPI networks [11,12]. Recently several studies also paid much attention to the association between PPI network topology and disease. For example, [13] found that inter-modular hubs are more associated with breast cancer than intra-modular hubs; [14] used pair-wise shortest paths between differentially expressed genes to identify candidate markers, [15] used probabilistic activity inference method to identify diagnostic subnetworks.

In this study we propose a network topology-based approach to identify candidate biomarker genes, motivated by the key observation that disease genes play a role in connecting differentially expressed (DE) genes in PPI networks [10]. For example, breast cancer biomarkers P53 and KRAS are not differentially expressed in metastatic breast cancer but they connect many DE genes in the human PPI network and play a central role in carcinogenic process [10]. The main idea of our approach is to find a small number of genes that can connect DE genes into a singly connected component in a PPI network, which maps to the well-known Steiner tree problem in graph theory and is solved using a heuristic algorithm. In addition, we combine multiple suboptimal Steiner trees to increase the chance of finding the optimal solution and to capture alternative pathways. Applying our approach on three breast cancer datasets, we found that the candidate markers selected by our method are highly enriched in pathways that are well-known to be dysregulated in breast cancer metastasis, and cover a significant number of known breast cancer susceptibility genes. Remarkably, the markers identified from multiple datasets have much higher reproducibility than in previous studies, and significantly increase the cross-datasets classification accuracy.

## Methods

### Datasets and PPI networks

In this study we used two microarray datasets herein referred as van de Vijver and Wang dataset [4,5] respectively. The two datasets have 295 and 286 breast cancer patients where 78 and 106 patients have distant metastasis within five years of follow-up visit respectively. The microarray platform used for van de Vijver et al was Agilent Hu25K and for other dataset was Affymetrix HG-U133a. The first dataset was downloaded from the Netherland Cancer Institute website (http://bioinformatics.nki.nl/index.php) while the other dataset was obtained from GEO with the accession number GSE2034 [16]. SAM (Significant Analysis of Microarray) [17] was used to select genes that are significantly differentially expressed between metastatic and non-metastatic tumors (DE genes). We controlled the delta parameter in SAM to select a similar number of DE genes from each dataset. As a result a total of 333 and 319 DE genes were selected for van de Vijver and Wang datasets, corresponding to FDR 0.7% and 8.2% respectively. Varying the number of DE genes between 200 and 1000 only slightly changed the percent of overlap while the significance of the overlap is essentially not affected (data not shown).

Two human protein-protein interaction networks were used by this study. The first network was obtained from Protein Interaction Network Analysis (PINA) and contains 10,920 genes and 61,746 binary connections [12]. The second network was compiled by [10] from six different sources, and contains 57,235 interactions among 11,203 genes. In this study we only considered the largest connected component in each PPI network, which contains 10,794 genes and 56,864 connections for Chuang PPI network and 10,770 genes and 61,658 connections for PINA PPI network.

### Randomized Steiner tree approach

**Figure 1:**
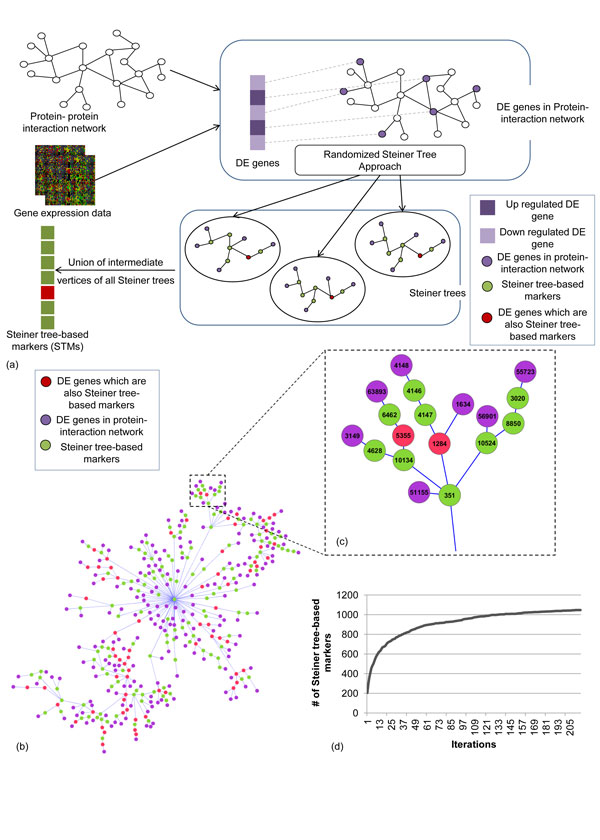
**Overview of our approach**. (a) A overview of our approach is given. Differentially expressed genes were selected from the dataset and then used with the protein protein interaction network to identify markers using Steiner tree approach. (b) A Steiner tree for van de Vijver dataset with Chuang PPI network is shown. (c) A larger figure for the highlighted part of the tree is shown. The ids of the vertices are in entrez gene id. (d) After 200-300 iterations the number of STM saturates to 1047 genes.

The main idea of our approach (see Figure 1) is to identify a small number of genes that connect all DE genes in a PPI network. In graph theory this problem is known as Steiner tree problem. The Steiner tree for an edge-weighted graph G= (V, E, w) and a subset of vertices R ⊆ V is a minimum-weight connected tree T, with vertices U ⊆ V and edges S ⊆ E that spans all vertices in R. Here the vertices in R are known as terminal vertices and U\R as Steiner vertices. For an unweighted graph G, the problem then becomes to find the minimum number of vertices that can connect all the vertices in R through a tree in G. The Steiner tree problem is NP-hard [18]. We implemented a slightly improved version of a polynomial-time 2-approximation shortest path heuristic algorithm [19]. This algorithm starts with a forest $\left(T^{{}^{\prime }}\right)$ comprising the terminal vertices R. Then in each iteration it finds the two vertices in $T^{{}^{\prime }}$ that are closest in distance and adds the intermediate vertices to $T^{{}^{\prime }}$. This procedure is repeated until $T^{{}^{\prime }}$ becomes connected. Finally, a minimum spanning tree of those selected vertices are built and all leaf vertices that are not in R are removed. The running time of this algorithm is *O*(|*V*|^3^). The pseudocode is given below.

### Steiner tree algorithm

**Input: **Weighted PPI network, *G *= *(V*,*E*,*w)*; DE genes, *R*

**Output: **Tree, *T*, that spans *R*

1. Start with a forest $\left(T^{{}^{\prime }}\right)$ comprising the DE genes, R, but no edges

2. **While **$T^{{}^{\prime }}$ is not a tree **do **Connect two shortest-distance disconnected vertices u, v ∈ $T^{{}^{\prime }}$ and add vertices on the path to $T^{{}^{\prime }}$

3. Build a minimum spanning tree (*T*) with the subgraph of G induced by the vertices in $T^{{}^{\prime }}$

4. Delete any leaf node in *T *that is not in *R*

In a Steiner tree the intermediate vertices (both Steiner vertices and non-leaf DE genes) play important roles in connecting DE genes together. We consider all the intermediate vertices as potential biomarkers (**S**teiner **T**ree-based **M**arkers or **STM**s) for breast cancer metastasis, as it is known that disease related genes play an important role in connecting DE genes in PPI network [10]. For example, for van de Vijver dataset with Chuang-PPI network, a single Steiner tree uses 136 vertices to connect 333 DE genes (Figure 1B-C). Among the DE genes, 264 are leaf nodes (degree = 1 in the tree) and the other 69 are internal nodes (degree > 1 in the tree). As these internal DE genes are important in connecting the remaining DE genes, we combine the Steiner vertices with these internal DE genes as potential biomarkers for breast cancer metastasis. Thus for this single tree we consider those 205 internal genes as potential biomarker (Figure 1B-C).

Next, we proposed a simple strategy to obtain multiple Steiner trees. The motivation is two-fold. First, as the heuristic algorithm does not guarantee optimality, by obtaining multiple solutions we increase the chance of finding the optimal Steiner vertices. Second, multiple solutions with similar qualities may represent alternative or redundant pathways that cannot be covered by a single Steiner tree. To obtain alternative Steiner trees without any modification to our Steiner tree algorithm, we assign to each edge in PPI network a random weight between 0.99 to 1 and run the standard Steiner tree algorithm. These random weights effectively break ties, so that if there are two paths with the same weight in the original network, one path will be chosen randomly. This procedure was repeated multiple times with different random weights from 0.99 to 1, until the total number of unique STMs converges approximately. Depending on the PPI network and microarray data, the rate of new coming STMs reduced significantly after 200-300 iterations (for example, see Figure 1D). After that, we take union of all internal nodes (or genes) of those trees and consider them as potential biomarkers. As previously mentioned, we called these genes as **S**teiner **T**ree-based **M**arkers (**STM**s). We obtained 1047 and 1100 STMs for Chuang PPI network and, 932 and 1135 STMs for PINA PPI network for van de Vijver and Wang dataset respectively (see Additional file 1 for complete gene list).

### Statistical test of overlap significance

Let N be the number of genes in the largest connected component of a PPI network, *m *and *n *the sizes of two gene sets, *o *the size of the overlap, the percent overlap between the two gene sets is calculated as 100 × *o*/(*m *+ *n *- *o*), and the statistical significance (p-value) of the overlap is calculated using Fisher's exact test:

(1) $$p=1-\overset{o-1}{\underset{i=0}{\sum }}C\left(m,i\right)C\left(N-m,n-i\right)/C\left(N,n\right),$$

where *C(n, k) *is the binomial coefficient.

### Classification

To evaluate the prediction ability of different features (STMs and DEs), we built logistic regression and support vector machine (SVM) classifiers to distinguish breast cancer patients who developed metastasis within five years after the date of the initial diagnosis from those who did not. We used the implementation in WEKA (version 3.6.3) and default parameter settings for this classification purpose [20]. To avoid overfitting and provide a realistic evaluation, we concentrated on cross-data classification where features obtained from one dataset were used to construct classifiers for the other dataset, because DEs were selected using the complete dataset and very specific to that particular dataset. Classification performance was estimated 100 times using 10-fold cross validation where iteratively one-tenth of the data were used for testing and nine-tenth were for training. Performance was measured by AUC (area under ROC curve).

## Results

### Stability of STMs

We first examined the stability of STMs across different datasets. For this we find the common genes in STMs for the two datasets with two PPI networks and compare with previous studies. The stability of STM and markers selected by previous studies are shown in Table 1. As can be seen, the overlap of STMs for the two datasets is 23.6% (p-value < 6.2E-159, Fishers' exact test) and 21.8% (p-value < 7.7E-138) for Chuang and PINA PPI respectively, while the overlap is 12.7% (p-value < 6E-54) in Chuang et al [10] and only 2% (p-value < 0.027) for the markers selected in the original studies [4,5]. The overlap between DE genes is 7.8% (p-value <1.3E-019). Thus for both networks the Steiner tree based markers have significantly better stability than the markers selected by other methods and the DE genes. Furthermore, the STMs selected from different datasets using different PPIs show similar level of stability as can be seen in Table 2.

**Table 1:** Stability of STMs

	van de Vijver dataset	Wang dataset	Number of common genes (% of overlap)	p-value
Chuang-PPI (STM)	1047	1100	410(23.6%)	6.16E-159
PINA-PPI (STM)	932	1135	370(21.8%)	7.67E-138
DE genes	333	319	47(7.76%)	1.32E-019
Result in [10]	618	906	175(12.7%)	5.57E-054
Result in [4,5]	70	76	3(2.09%)	0.0274

The percentage of overlap and p-values between van de Vijver and Wang datasets in different methods are given.

**Table 2:** Cross PPI stability of STMs

	van de Vijver dataset	Wang dataset	Number of common genes (% of overlap)	p-value
Chuang PPI vs PINA PPI	1047	1135	306(16.6%)	6.67E-072
PINA-PPI vs Chuang PPI	932	1100	283(16.2%)	2.14E-073
DE genes	333	319	47(7.76%)	1.32E-019

The percentage of overlap and p-values between cross datasets and cross PPI network.

### Functional enrichment and pathway analysis of STMs

**Figure 2:**
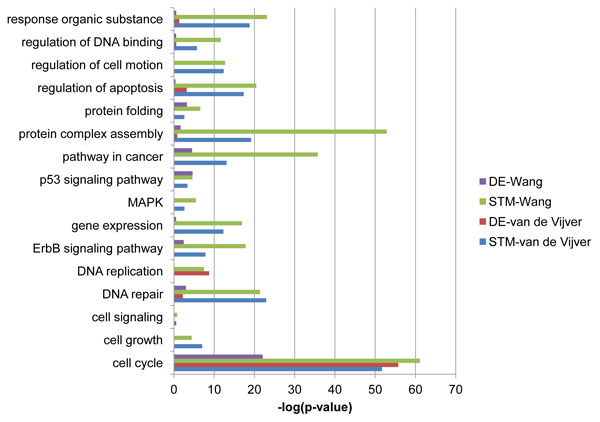
**Enrichment of STMs and DEs**. Enriched biological processes and pathways of STMs and DE genes for van de Vijver and Wang datasets for Chuang PPI network is shown. PINA PPI shows similar results.

To reveal the biological functions of the candidate biomarkers we used online bioinformatics resource DAVID [21] to analyze the enriched gene ontology biological processes and KEGG pathways for STMs and DEs. We used the genes in the largest connected component of the PPI network as the background gene list in DAVID. This analysis showed that genes selected by our method are significantly enriched in functional terms that are well known to be involved in cancer carcinogenic process, including cell cycle, apoptosis, DNA repair as well as MAPK, ErbB and P53 signaling pathways. A list of the enriched terms and their enrichment scores are listed in Figure 2. These enriched functional terms and pathways have strong consistency with several previous studies [10,22,23]. DE genes clearly have much lower significance compared to STMs. For example, for van de Vijver dataset and Chuang PPI network DE genes are only weakly enriched in DNA repair (p-value<1.1E-1 vs 1.1E-10 in STMs) and apoptosis (p-value<4.2E-2 vs 3.0E-8 in STMs), and not significantly enriched in cell growth, ErbB, MAPK and P53 signaling pathways. A similar trend is found in Wang dataset (Figure 2). Using PINA PPI network for these two datasets we also have similar and significant results for STMs compared to DE genes (see Additional file 2). Thus starting with the DE genes with low significance our method is able to find a set of genes which are overrepresented in many known pathways involved in breast cancer metastasis.

### STMs correspond to novel biomarkers of cancer

**Figure 3:**
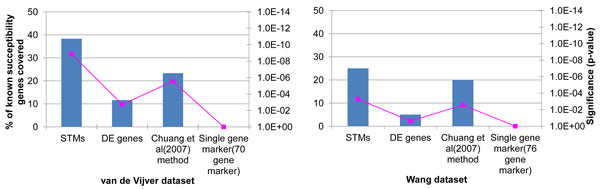
**Known breast cancer susceptibility genes covered by STMs**. Overlap of know breast cancer susceptibility genes with STMs, DEs and genes selected from previous studies are shown.

Next, to demonstrate that our method is able to identify cancer susceptibility genes, we compare our results with Chuang et al [10]. In that study they collected 60 known breast cancer marker genes from Online Mendelian Inheritance in Man (OMIM). As shown in Figure 3, for both datasets STMs have higher percentage of overlaps with those known markers than the genes selected by previous studies [4,5,10]. Also, DE genes cover a smaller portion of known cancer susceptibility genes than STMs. The enrichment of known breast cancer markers in STMs (p-value < 1.32E-09 and 5.28E-04, for van de Vijver and Wang datasets respectively, Fishers' exact test) are also more significant than in DE genes and genes selected by previous studies [4,5,10]. Some well-known biomarkers covered by STMs are BRCA1 (breast cancer 1, early onset), BRCA2 (breast cancer 2, early onset), ATM (Ataxia telangiectasia mutated), TP53 (tumor protein p53), ErbB2 (a.k.a. HER2, Human Epidermal Growth Factor Receptor 2), TFN (tumor necrosis factor) and Esr1 (Estrogen receptor 1) (for a complete list see Table 3). As the STMs are enriched in breast cancer related pathways and cover a significant number of known susceptibility genes, the rest of the STMs that are not known biomarkers can be considered as novel potential biomarkers for breast cancer metastasis.

**Table 3:** Breast cancer genes in STMs

Breast cancer known genes in STMs (van de Vijver dataset)	Breast cancer known genes in STMs (Wang dataset)
*RAD54L*	*HRAS*
*HRAS*	*ITGA2*
*ERBB2*	*BRCA2*
*BRCA2*	*BRCA1*
*PGR*	*APC*
*XRCC1*	*KRAS*
*BRCA1*	*ITGB3*
*PHB*	*ESR1*
*TYMS*	*TP53*
*TNF*	*TGFB1*
*APC*	*VDR*
*ESR1*	*AR*
*TP53*	*RAD51*
*PIK3CA*	*TSG101*
*TGFB1*	*CDH1*
*GSTP1*	
*GSTT1*	
*LOC651610*	
*ATM*	
*RAD51*	
*TK1*	
*CYP1A1*	
*TSG101*	
*CHD1*	

Known breast cancer susceptibility genes covered by STMs from van de Vijver and Wang datasets for Chuang PPI network.

We also collected 288 breast cancer susceptibility genes from Genetic Association Database of Disease from DAVID. STMs covered 26.39% and 26.04% of those known breast cancer susceptibility genes for Wang and van de Vijver datasets, respectively. For both datasets, genes selected by our method clearly outperformed DE genes (5.55% and 5.28% for Wang and van de Vijver datasets respectively). Evaluation results using PINA PPI show similar results.

### Steiner tree-based markers improves the cross-dataset classification accuracy

**Figure 4:**
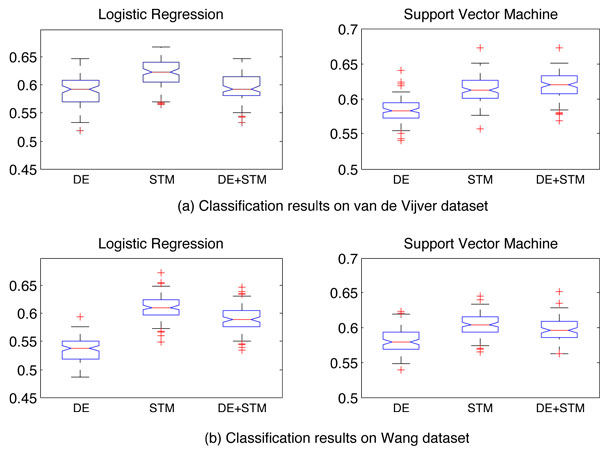
**Classification results**. Classification accuracy of DE and STM genes based on AUC metric with logistic regression and Support Vector Machine are shown. The dataset label represents features taken from the other dataset and applied to the labeled dataset.

We tested whether the STMs can be used to improve the prediction accuracy of breast cancer metastasis. To this end we use the STMs as features to train logistic regression and support vector machine (SVM) classifiers [20,24] to separate metastatic from non-metastatic patients (see method section). For comparison, we also constructed classifiers using only DE genes and DE+STM genes. To evaluate the power of the candidate markers in predicting metastasis in an unbiased fashion, we focused on cross-dataset tests, where the genes selected from one dataset were used to construct classifiers for the other dataset. Figure 4 shows the classification accuracy, measured by AUC (area under ROC curve), achieved by different feature sets for logistic regression and support vector machine classifier. As can be seen, STMs resulted in better accuracy than DEs for both cases. On van de Vijver dataset, STM-based logistic regression classifier performed better than DE-based classifiers in 79 of the 100 runs (p-value = 4E-12, paired t-test), and STM-based SVM classifier outperformed DE-based SVM in 87/100 of runs (p-value = 6E-20). For Wang dataset, the corresponding numbers are 99/100 (p-value = 7.7E-44) and 86/100 (p-value = 5.5E-17). On the other hand, STMs performed better than STM+DE feature set in all cases except in van de Vijver dataset for SVM classifier. In that case STM+DE sets performed slightly better than the STMs. For all cases STM+DE set performed better than DEs. Thus STMs have better classification accuracy on predicting breast cancer metastatic potentials than DE genes.

Next we compared our classification results with [10]. In table 4 the classification results for [10] and our method are summarized. From the table it can be seen that for van de Vijver dataset the percentage of increase of AUC for [10] is 7.5% based on their subnetwork markers compared to single gene markers (DEs). For our case we observe a 5% increase of AUC for STMs compared to DEs. For Wang dataset [10] subnetwork markers have 3% increase in AUC while STMs have 15% increase in AUC. For the overall classification accuracy, STMs have similar discriminative power as [10] in Wang dataset and slightly lower accuracy for van de Vijver dataset. This deviation may be due to the fact that [10] used feature selection for classification purpose and we used all selected genes to build the classifier. For this case feature selection may increase classification accuracy which not necessarily reflects the actual discriminative power of the overall selected genes by a certain method. As our main focus is to identify biomarkers and to see their overall classification performance to validate the reproducibility across different datasets, classification model that contain the overall features (genes) selected by our method(STMs) is a valid way for doing that.

**Table 4:** Classification accuracy of STMs

	**Chuang et al **[10]**method**			Steiner tree based method		
	Single gene marker	Subnetwork marker	% increase of AUC	DEs	STMs	% increase of AUC
van de Vijver dataset	0.67	0.72	7.5%	0.59	0.62	5%
Wang dataset	0.6	0.62	3%	0.53	0.61	15%

Comparison of classification results with [10] method.

## Conclusions

In this article we proposed a randomized Steiner tree-based approach that integrates a PPI network and gene expression microarray data for biomarker discovery in breast cancer metastasis. The genes selected by our method are significantly enriched in functional categories and pathways that are known for cancer development. Furthermore, a significant portion of selected genes by our method are already known for breast cancer susceptibility. We applied the method to three different breast cancer microarray data and two different PPI networks. For all combinations of microarray and PPI datasets our approach has similarly significant results. The reproducibility across different datasets also increases significantly in both genomic and pathway level compared to previous studies. Finally, Steiner tree-based markers, significantly increase cross-dataset classification accuracy. Thus the method proposed in this article validates the hypothesis that disease causal genes play a role in connecting differentially expressed genes, and opens a new possibility to identify the inner dynamics and biomarker of breast cancer progression.

## Abbreviations

PPI: protein protein interaction; DE: differentially expressed; STM: Steiner tree-based marker; ROC: receiver-operating characteristic; AUC: area under ROC curve.

## Competing interests

The authors declare that they have no competing interests.

## Authors' contributions

JR conceived of the research. MJJ and JR designed the study. MJJ did the experiments. MJJ and JR wrote the manuscript.

## Supplementary Material

Additional file 1 **This excel file contains the entrez ids of the Steiner tree-based markers (STMs) selected by this approach for van de Vijver and Wang datasets using Chuang and PINA PPI networks**. Besides this, differentially expressed (DE) genes for the two datasets are also given. Click here for file

Additional file 2 **This pdf file contain the figure of enrichment of STMs and DEs**. Enriched biological processes and pathways of STMs and DE genes for van de Vijver and Wang datasets for PINA PPI network is shown here. Click here for file
