# Supplementary figures and images for: A Steiner tree-based method for biomarker discovery and classification in breast cancer metastasis
Source: BMC Genomics. 2012 Oct 26;13(Suppl 6):S8. doi: 10.1186/1471-2164-13-S6-S8 (PMC3481447; doi:10.1186/1471-2164-13-S6-S8)

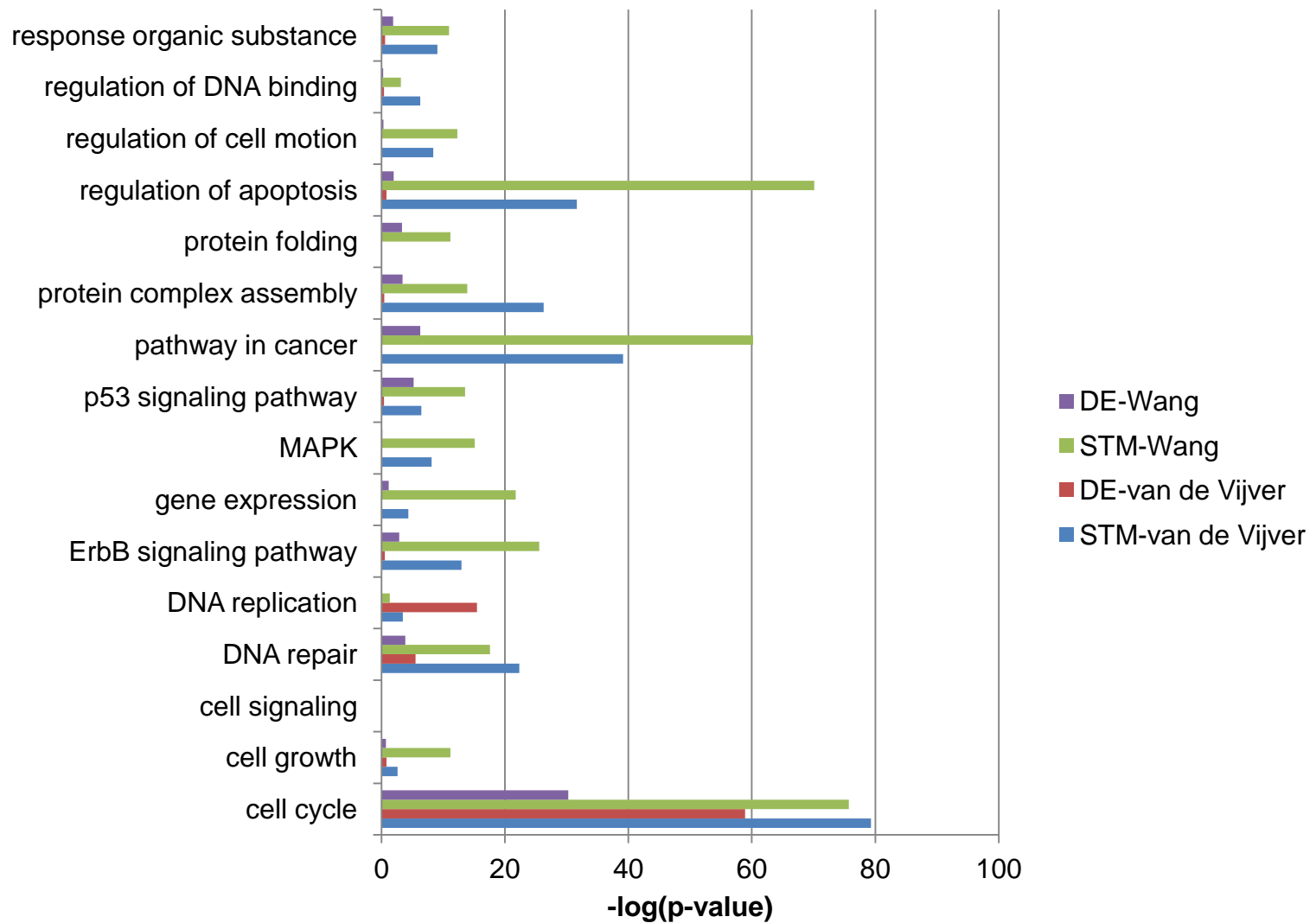

Supplement: Additional file 2 — This pdf file contain the figure of enrichment of STMs and DEs. Enriched biological processes and pathways of STMs and DE genes for van de Vijver and Wang datasets for PINA PPI network is shown here. [file 1471-2164-13-S6-S8-S2.pdf]
